# Blindness and visual impairment due to retinal diseases

**Published:** 2009-03

**Authors:** Shaheen Shah

**Affiliations:** Clinical Research Fellow, International Centre for Eye Health, London School of Hygiene and Tropical Medicine, Keppel Street, London WC1E 7HT, UK.

**8^th^ General Assembly of IAPB**

**Symposium 8:** Diabetic retinopathy

**Speakers:** Anthony Hall, David Yorston, Juan Carlos Silva, RD Ravindran

**Plenary 2:** Emerging priorities

**Speakers:** Hugh R Taylor, Serge Resnikoff, RD Ravindran, Hasan Minto, Babar Qureshi, Santiago Castro Feijo

**Course 18:** Vitreoretinal services

**Speakers:** Anthony Hall, David Yorston, Pedro Gomez, Marcelo Ventura

**Figure:**
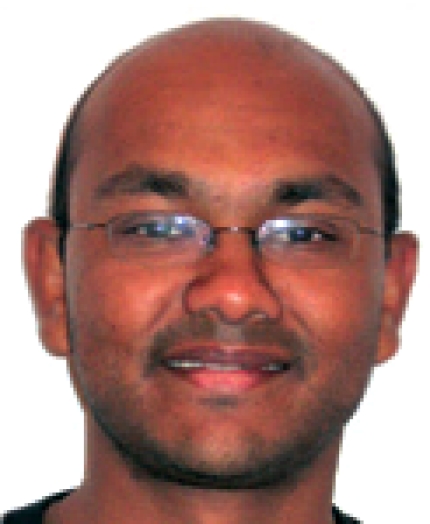


This General Assembly, compared to others in the past, had retinal diseases as a cause of visual impairment and blindness higher up on the agenda. This article summarises the main points made in relation to retinal diseases, in particular the challenge of diabetic retinopathy.

## General overview on retinal diseases

### A global epidemic of diabetes mellitus

Diabetes mellitus is a chronic disease that occurs when the pancreas does not produce enough insulin, a hormone that regulates blood sugar. Diabetes may be insulin-dependent, which is typically of early onset, or non insulin-dependent, when the body cannot effectively use the insulin it produces (maturity onset diabetes). Hyperglycaemia, or raised blood sugar, is a common effect of uncontrolled diabetes and over time leads to serious damage to many of the body's systems, especially the eyes, kidneys, and nerves.^1^

The world is currently experiencing a global epidemic of this incurable disease. Current predictions estimate a doubling of the number affected from the current 171 million to an estimated 366 million by 2030 (Table 1). Global obesity maps presented at the meeting similarly showed a striking increase all over the world. The global increase of diabetes is attributed to increased life expectancy, urbanisation, and a change in life style and diet.

**Table 1:** Number of people with diabetes (in millions): top ten countries^1^

Country	2000	2030 (predicted)
India	31.7	79.4
China	20.8	42.3
USA	17.7	30.3
Indonesia	8.4	21.3
Japan	6.8	8.9
Pakistan	5.2	13.9
Russia	4.5	5.3
Brazil	4.6	11.3
Italy	4.3	5.3
Bangladesh	3.1	11.1
Worldwide	171	366

**Figure:**
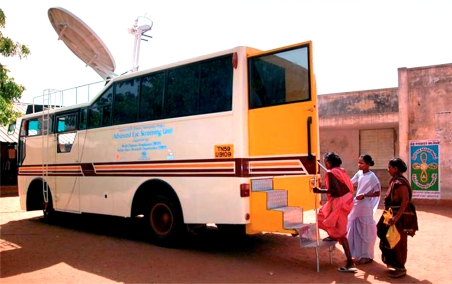
Mobile screening van for diabetic retinopathy. INDIA

RD Ravindran

Although previously recognised as a disease of the developed world, diabetes is becoming increasingly prevalent in the developing world and the majority of the burden from this disease is predicted to affect people of working age in low-income countries.^2^^,^^3^ In India alone, the number affected is expected to reach 80 million by 2030. As a shocking example, RD Ravindran cited the case of a 57-year-old physician who had had diabetes for 16 years and who presented with severe proliferative retinopathy. This highlighted not only a general lack of awareness, but also the fact that diabetic retinopathy may not be considered by some physicians to be an important complication.

#### Ocular complications of diabetes:

The main microvascular complication of diabetes in the eye is diabetic retinopathy (DR), which is found in almost 20% of newly diagnosed diabetic people.

It is important to remember that diabetes is also a risk factor for cataract, so the retinopathy may only be evident once the cataract has been removed and a view of the posterior segment is possible.^4^

Diabetic retinopathy is increasing as a cause of blindness throughout the world and data presented at the meeting suggests that DR accounts for an estimated 5% of the 45 million blind people worldwide today.

### Age-related macular degeneration (AMD)

AMD was another retinal disease discussed and highlighted as a growing concern. It is a disorder predominantly affecting people over the age of 60. It affects the central part of the vision, which is essential for detailed tasks requiring fine vision, such as reading and recognising faces.

By 2025, there will be twice as many older people worldwide as there were in 2000 (an increase from 606 million to 1.2 billion). Twenty-fve years later, by 2050, the population of older people will be three times greater than in 2000, around two billion,^5^ and subsequently the number of people with AMD will increase significantly.

Figures presented by Serge Resnikoff suggest that AMD is already the third largest cause of blindness in the world, as it accounts for 9% of the 45 million blind people worldwide.

#### Treatment:

Unfortunately, although new and exciting treatment strategies have been demonstrated to be beneficial in the active ‘wet’ form of AMD (intravitreal injections of anti-vascular endothelial growth factor), they require repeated administration, are not curative, and are prohibitively expensive.

On the other hand, smoking is an established risk factor for AMD and tobacco control should be strongly advocated as a measure for the prevention of blindness. For example, in Australia, cigarette packets display a prominent warning saying: ‘Smoking causes blindness’. Currently, smokers represent 25% of the worldwide population. Resnikoff explained that, if this figure were to fall from 25% to 15%, this would avoid 100,000 cases of blindness. Unfortunately, figures presented at the meeting show that, as the tobacco industry reaches new markets, the share of the worldwide consumption of cigarettes represented by developing countries is steadily increasing. Currently, developing countries account for approximately 70% of global tobacco consumption.

### Retinal detachment (RD)

The annual incidence of RD is estimated at 10/100,000 per year, although there are regional variations. It was roughly estimated that, globally, 90 eyes are blinded by RD every hour.

Risk factors for RD are increasing age, myopia, and cataract surgery. All these risk factors are becoming more common.

Results from low-income countries show that many patients present only when they lose vision in both eyes. Delay in presentation was acknowledged as a significant problem in the management of RD. It is often due to inadequate primary eye care and to misdiagnosis. Thus, it was highlighted that all ophthalmologists should be trained to recognise and manage RD appropriately.

## Secondary prevention: screening for diabetic retinopathy

As blindness from DR is preventable, if caught and treated early (before symptomatic visual loss), DR provides an excellent opportunity for secondary prevention strategies, such as screening.

A number of different screening models were discussed at the meeting.

### Scotland

In Scotland, 24% of the population is obese — a figure second only to the US — and it is estimated that 3–4% of the population has diabetes. The model used for the national screening programme was a system of ‘gatekeepers’ on three levels, each with increasing expertise: a trainee screener (e.g. a nurse) assessed the presence or not of retinopathy, then a trained screener (e.g. an optometrist) assessed whether the patient with retinopathy needed to be referred for laser treatment, and this was confirmed by an ophthalmologist at the third level of screening.

However, this system is extremely costly and probably difficult to replicate in low- or middle-income countries. The automated grading of fundus photographs appears to be an exciting development, as it can reduce costs. One speaker mentioned a project comparing the cost-effectiveness of manual and automated grading for DR. It showed that, although manual grading was more accurate, it cost UK £4,000 (US $5,750) per additional case detected.^6^

**Figure:**
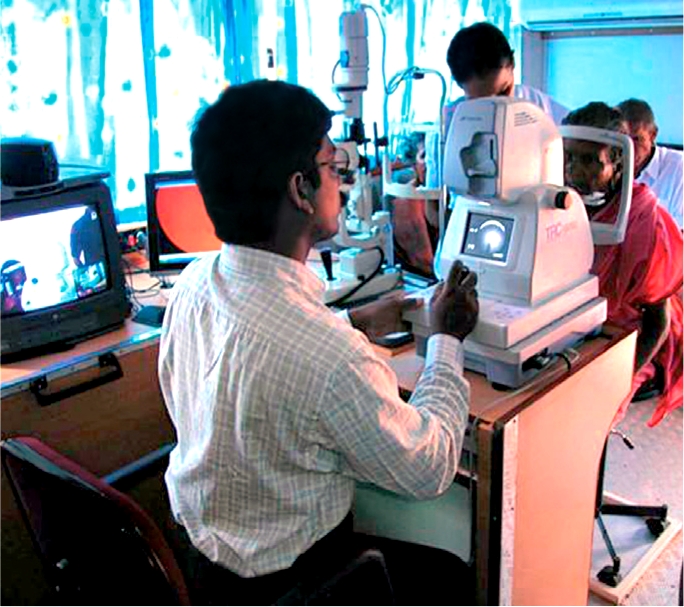
Fundus examination inside a screening van. INDIA

RD Ravindran

### India

The Aravind model in India uses a number of different methods to screen for DR. These range from screening for diabetes in the general population (nearly 230,000 screened) and then screening the suspects for DR, to using telemedicine facilities for patients known to have diabetes.

#### Telemedecine:

Telemedicine was advocated as a tool to improve rural eye care, as there is a disparity in the distribution of eye care resources. Two applications were described:

- a mobile model using a van with satellite connection to the Internet
- a physician- and clinic-based model using a fixed broadband connection.

Both models operate in two modes:

- real time or interactive videoconferencing
- store and forward to base hospital later.

Software designed for use within the screening process allows automatic generation of reports with possible treatment strategies. The results presented showed that nearly 52,000 people were screened using the mobile method and DR was found in 1,300, of which 76% had retinopathy detected for the first time.

### Latin America and the Caribbean

In Latin America, there has been no adequate situational analysis and there is limited logistic and technical skills.

On the Caribbean island of Dominica, the DR screening programme utilises an itinerant ophthalmic technician with a digital fundus camera. Only 35% of the target population was screened in 2007 and, furthermore, compliance for referral was poor (44%).

The figures were better in Medellin, Colombia, where nearly 70% of people known to have diabetes attended screening. Third-year trained residents graded the photographs. Retinal specialists provided treatment and treatment rates were high, with 25% of those found to have DR at screening receiving laser treatment.

### Offering screening and treatment hand in hand

A particular concern expressed by all speakers and highlighted as a significant barrier was the practice of setting up a screening system without adequate treatment facilities being in place. Laser treatment is largely unavailable in many low-income countries. Even when it is available, it is often inaccessible and expensive.

## Tertiary vitreoretinal services

A new theme this year was the establishment of tertiary level vitreoretinal (VR) services. VR services (which offer predominantly pars plana vitrectomy) treat the complications of severe DR and/or patients with a retinal detachment (RD).

Primary success of vitrectomy in low-income countries averaged 60–80% and nearly 60% of patients achieved a visual acuity of 6/60 or better after surgery.

Vitrectomy also has a place in the treatment of one of the serious complications of phacoemulsification cataract surgery — a ‘dropped’ nucleus.

Training in this speciality is a difficult issue and it was argued that a training unit in VR needs to be performing at least 200 retinal cases a year.

## Integration with other health sectors

Finally, all the speakers mentioned that services for retinal pathology needed to be better integrated with other health care sectors. This was a recurrent theme of the meeting.

Services are currently disjointed; for example, orthopaedic doctors perform leg amputations in patients with diabetes, but they do not refer these patients for an eye examination.

Speakers advocated a better integration of blindness prevention strategies into national diabetes programmes.

**Diabetic retinopathy: key points**

1. There is a need for **better public education and awareness** (through community stakeholders and media).
2. There should be **better coordination** between eye care personnel and other health care personnel (e.g. eye teams should liaise with physicians in health centres).
3. **Diabetes registers** should be maintained and kept up to date.
4. **Barriers to the use of services** should be identified. For example, in Tanzania, although the check up was free, one-fifth of diabetic patients left the eye department after being given dilating drops and before being examined, because the waiting time was too long.
